# Identification of significant alteration genes, pathways and TFs induced by LPS in ARDS via bioinformatical analysis

**DOI:** 10.1186/s12879-021-06578-7

**Published:** 2021-08-21

**Authors:** Weina Lu, Ran Ji

**Affiliations:** Department of Surgical Intensive Care Unit, Second Affiliated Hospital, Zhejiang University School of Medicine, No. 88 Jiefang Road, Hangzhou, 310000 China

**Keywords:** Acute respiratory distress syndrome, Bioinformatical analysis, Differentially expressed gene, Microarray

## Abstract

**Background and aims:**

Acute respiratory distress syndrome (ARDS) or acute lung injury (ALI) is one of the most common acute thoracopathy with complicated pathogenesis in ICU. The study is to explore the differentially expressed genes (DEGs) in the lung tissue and underlying altering mechanisms in ARDS.

**Methods:**

Gene expression profiles of GSE2411 and GSE130936 were available from GEO database, both of them included in GPL339. Then, an integrated analysis of these genes was performed, including gene ontology (GO) and KEGG pathway enrichment analysis in DAVID database, protein–protein interaction (PPI) network construction evaluated by the online database STRING, Transcription Factors (TFs) forecasting based on the Cytoscape plugin iRegulon, and their expression in varied organs in The Human Protein Atlas.

**Results:**

A total of 39 differential expressed genes were screened from the two datasets, including 39 up-regulated genes and 0 down-regulated genes. The up-regulated genes were mainly enriched in the biological process, such as immune system process, innate immune response, inflammatory response, and also involved in some signal pathways, including cytokine–cytokine receptor interaction, Salmonella infection, Legionellosis, Chemokine, and Toll-like receptor signal pathway with an integrated analysis. GBP2, IFIT2 and IFIT3 were identified as hub genes in the lung by PPI network analysis with MCODE plug-in, as well as GO and KEGG re-enrichment. All of the three hub genes were regulated by the predictive common TFs, including STAT1, E2F1, IRF1, IRF2, and IRF9.

**Conclusions:**

This study implied that hub gene GBP2, IFIT2 and IFIT3, which might be regulated by STAT1, E2F1, IRF1, IRF2, or IRF9, played significant roles in ARDS. They could be potential diagnostic or therapeutic targets for ARDS patients.

**Supplementary Information:**

The online version contains supplementary material available at 10.1186/s12879-021-06578-7.

## Introduction

Acute respiratory distress syndrome (ARDS) or acute lung injury (ALI) is an acute hypoxemic respiratory failure, characterized by lung tissue oedema and injury, inflammatory responses, and compromised gas exchange following macrophage activation, surfactant dysfunction, and epithelial destruction [1, 2]. It has been widely recognized as a clinical problem worldwide, accompanied by high morbidity and mortality [3, 4]. According to a recent international multi-centre research in 50 countries, the prevalence of ARDS was 10.4% of ICU admissions [5]. According to a cross-sectional study, the mortality of ARDS from 2012 to 2013 in Chinese 20 ICUs is about 34% [6]. Especially in the last 2 years, the incidence and mortality of COVID-19-associated ARDS have worsened the outcome. For the patients of COVID-19 ARDS in ICU, mortality ranged between 26 and 61.5%, especially for those who received mechanical ventilation, the mortality ranged between 65.7 and 94% [7]. Despite a variety of basic and clinical research held, there is still no effective pharmacotherapy for it. Currently, the treatment remains primarily with ventilation and conservative fluid management. Therefore, it is critical to study ARDS’s pathogenesis and explore specific biomarkers for this condition.

The development process of ARDS is complicated, and the specific mechanism is not yet fully understood. Multiple studies have confirmed that ARDS is related to the damage and disruption of the epithelial and endothelial cells, as well as dysregulated inflammation [8–10]. A breakdown in endothelial junctions or the injury of endothelial cells can aggravate lung vascular permeability. Cheng et al. [11] reported that the severe endothelial pyroptosis caused by bacterial endotoxin lipopolysaccharide (LPS) was mediated by the inflammatory caspases. IL-1β could impair CREB-mediated VE-cadherin transcription to induce endothelial injury [12]. Besides, lung epithelial permeability alteration is also an important factor in ARDS pathogenesis. Short et al. [13] showed that the alveolar barrier could be damaged by influenza by disrupting epithelial cell tight junctions, specifically with loss of tight junction protein claudin-4. Related gene mutations or expression alterations in the ARDS might be suitable to serve as diagnostic or therapeutic targets. Microarrays have been used to quantify the high-throughput expression of genes for many species quickly [14]. As a result, the data produced from microarrays were stored in some public databases. We could explore lots of valuable clues from these raw data for further experimental research. Some different bioinformatic studies have been exploited in the past few years, which provided us with abundant integrated bioinformatical methods for studies [15].

To identify the better potential diagnosis or therapeutic targets for ARDS, firstly, we performed a transcriptome analysis of mice lung tissues. The tissues were treated with LPS and the raw data was acquired from Gene Expression Omnibus (GEO) to explore differentially expressed genes (DEGs) and pathways. Once characterized hub genes, we could evaluate their expression in human lung tissues. Finally, the hub DEGs above mentioned were processed further to find the common TFs.

## Methods

### Approach

In this study, GSE2411 [16] and GSE130936 [17] profiles were chosen from Gene Expression Omnibus (GEO). Titles associated with ARDS or ALI were screened, and the details of these datasets, like organisms and samples, were further evaluated. In the next, the GEO2R online tool and Venn diagram software were applied to find the common differentially expressed genes (DEGs) in the two datasets. Then, we used the DAVID database to analyze these DEGs including molecular function (MF), cellular component (CC), biological process (BP), and Kyoto Encyclopedia of Gene and Genome (KEGG) pathways. Next, the protein–protein interaction (PPI) network with MCODE plugin was constructed. GO analysis and KEGG analysis were re-utilized to screen the hub genes, followed by the hub DEGs imported into the Human Protein Atlas database to evaluate their expression level in varied organs. At last but not least, the hub DEGs were processed by iRegulon to find the common TFs.

### Microarray data information

The relevant gene profiles were obtained from NCBI-GEO (https://www.ncbi.nlm.nih.gov/geo/), a public online database. Titles associated with ARDS were screened, and the details of these datasets were further evaluated. Two datasets were obtained at last, including GSE2411 and GSE130936. Microarray data of GSE2411 and GSE130936 were on account of GPL339 Platforms ([MOE430A] Affymetrix Mouse Expression 430A Array). Dataset GSE2411 included 6 wildtype mice control, and 6 wildtype mice injected intraperitoneally with LPS to induce experimental ARDS. Samples were obtained from the pulmonary tissues. They were marked from GSM45427 to GSM45432 and from GSM45439 to GSM45444, respectively. Dataset GSE130936 included wild-type mice that were induced either with saline as control (n = 4) or LPS (n = 3). Samples were labelled from GSM3756516 to GSM3756518 and GSM3756522 to GSM3756525 for further processing (Additional file 1).

### Data processing and DEGs identification

DEGs between ARDS pulmonary tissues and normal pulmonary tissues were identified by online tool GEO2R (https://www.ncbi.nlm.nih.gov/geo/geo2r/), with |logFC|> 2 and adjust *P* value < 0.05[18]. Then, the raw data were input in Venn software online (http://bioinformatics.psb.ugent.be/webtools/Venn/) to find the common DEGs between the two datasets [19]. At last, the genes in the common datasets with logFC > 0 were considered up-regulated genes, while those with logFC < 0 were considered down-regulated genes.

### Gene function and pathway enrichment analysis of DEGs

Gene ontology (GO) is a systematical approach for gene annotation, RNA and protein expression [20]. KEGG is an online database of genomes, enzymatic pathways, and biochemicals. The pathway database of KEGG records molecular interaction networks in cells and changes specific to specific organisms [21]. DAVID (https://david.ncifcrf.gov/) is a biological information database that integrates biological data and analysis tools to provide systematic comprehensive biological function annotation information for the large-scale gene or protein lists, helping us to extract from them biological information [22]. In this study, the DAVID database was used to perform GO analysis and KEGG pathway enrichment analysis in helping classifying DEGs (*P*-value < 0.05).

### PPI network construction of DEGs and significant module screening

Visualized protein–protein interaction (PPI) information of DEGs was evaluated by STRNG (https://string-db.org/) [23], an online database set for retrieving interacting genes. Subsequently, the result from STRING was imported into Cytoscape software to examine the potential correlation among these DEGs (maximum number of interactors = 0 and confidence shub ≥ 0.4) [24]. Lastly, MCODE plugin of Cytoscape was utilized to screen the obvious submodules and hub genes in the PPI network (degree cutoff = 2, k-hub = 2, node shub cutoff = 0.2 and the normalized enrichment shub (NES) > 12 [25]).

### Expression of hub genes in different human normal organs

The Human Protein Atlas (https://www.proteinatlas.org/) was a public database of the gene expression profile in human varied organs. The basic RNA and protein expression levels of specific genes could be identified from it. There were three RNA expression databases in the Human Protein Atlas, including the HPA dataset, the genotype-tissue expression (GTEx) project dataset and the Functional Annotation of the Mammalian Genome (FANTOM5) dataset. In this study, GTEx database was used to evaluate the hub genes’ mRNA expression level in different organs, particularly in the pulmonary tissue.

### Prediction of transcriptional factors (TFs) of hub genes

The Cytoscape plugin iRegulon was used to analyze transcription factors regulating the hub genes [25, 26]. The iRegulon plugin can identify regulons using motifs and track discovery in an existing network or a set of regulated genes. The cutoff criteria were as follows: enrichment shub threshold = 3.0, ROC threshold for AUC calculation = 0.03, rank threshold = 5000, minimum identity between orthologous genes = 0.0 and FDR = 0.001 [25].

## Results

### Identification of DEGs in ARDS

**Fig. 1:**
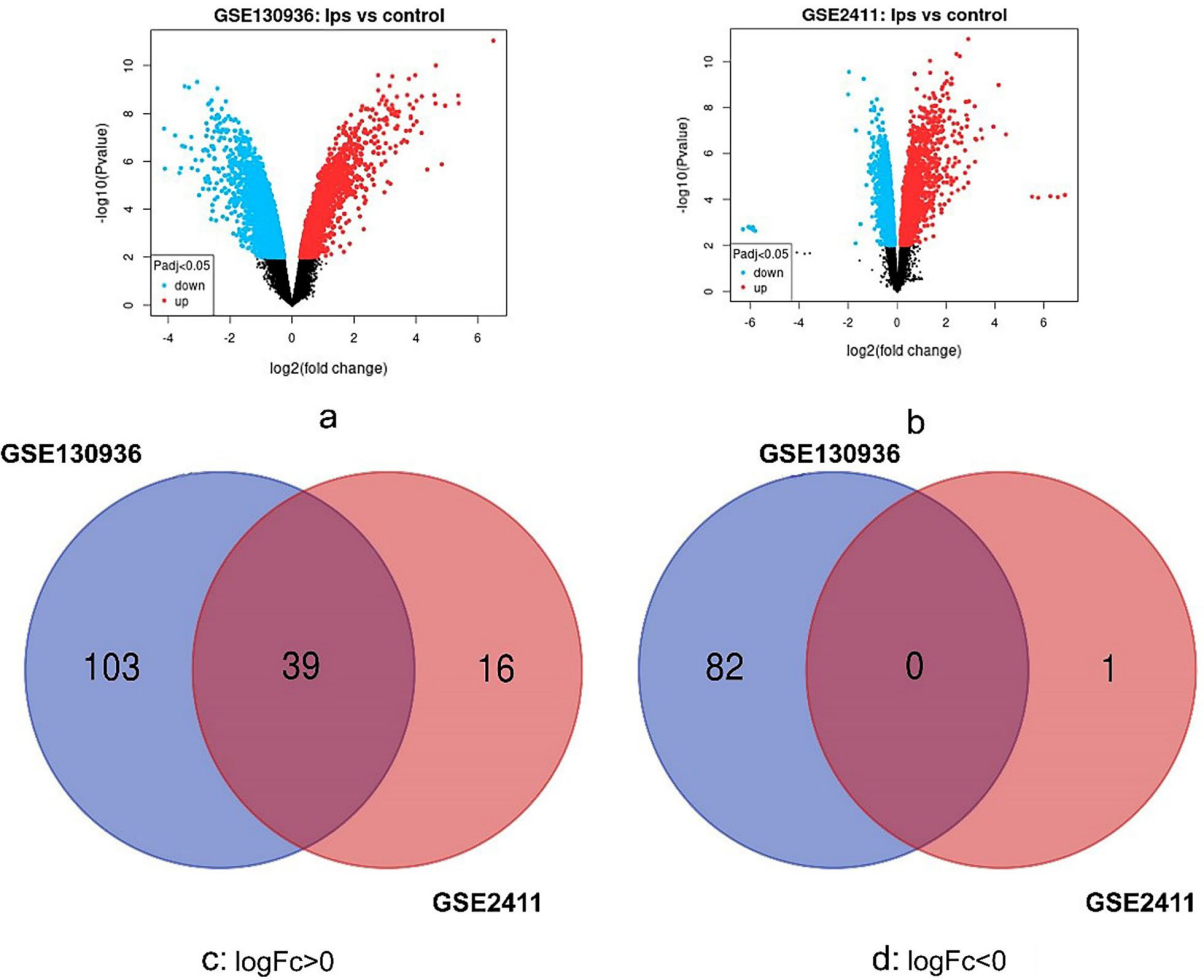
**a**,** b** Red meant up-regulated genes and blue mean downregulated genes, in GSE2411 and GSE130936 respectively. Authentication of 39 common DEGs in the two datasets (GSE2411 and GSE130936) through Venn diagrams software (available online: http://bioinformatics.psb.ugent.be/webtools/Venn/). Different color meant different datasets. **c** 39 DEGs were up-regulated in the two datasets (logFC > 0). **d** 0 DEGs were down-regulated in two datasets (logFC < 0)

Raw data of the microarray datasets from GEO datasets were processed by the CEO2R online tool. We extracted 224 and 56 DEGs from GSE130936 (https://www.ncbi.nlm.nih.gov/geo/geo2r/?acc=GSE130936) and GSE2411 (https://www.ncbi.nlm.nih.gov/geo/geo2r/?acc=GSE2411), respectively. Then, the common DEGs in the two datasets were identified by Venn diagram software. 39 common DEGs were obtained, including 39 up-regulated genes (log FC > 2, adjust *P* < 0.05) and 0 down-regulated genes (log FC < − 2, adjust *P* < 0.05) in the pancreas (Fig. 1).

### DEGs gene ontology and KEGG pathway analysis in ARDS

**Fig. 2:**
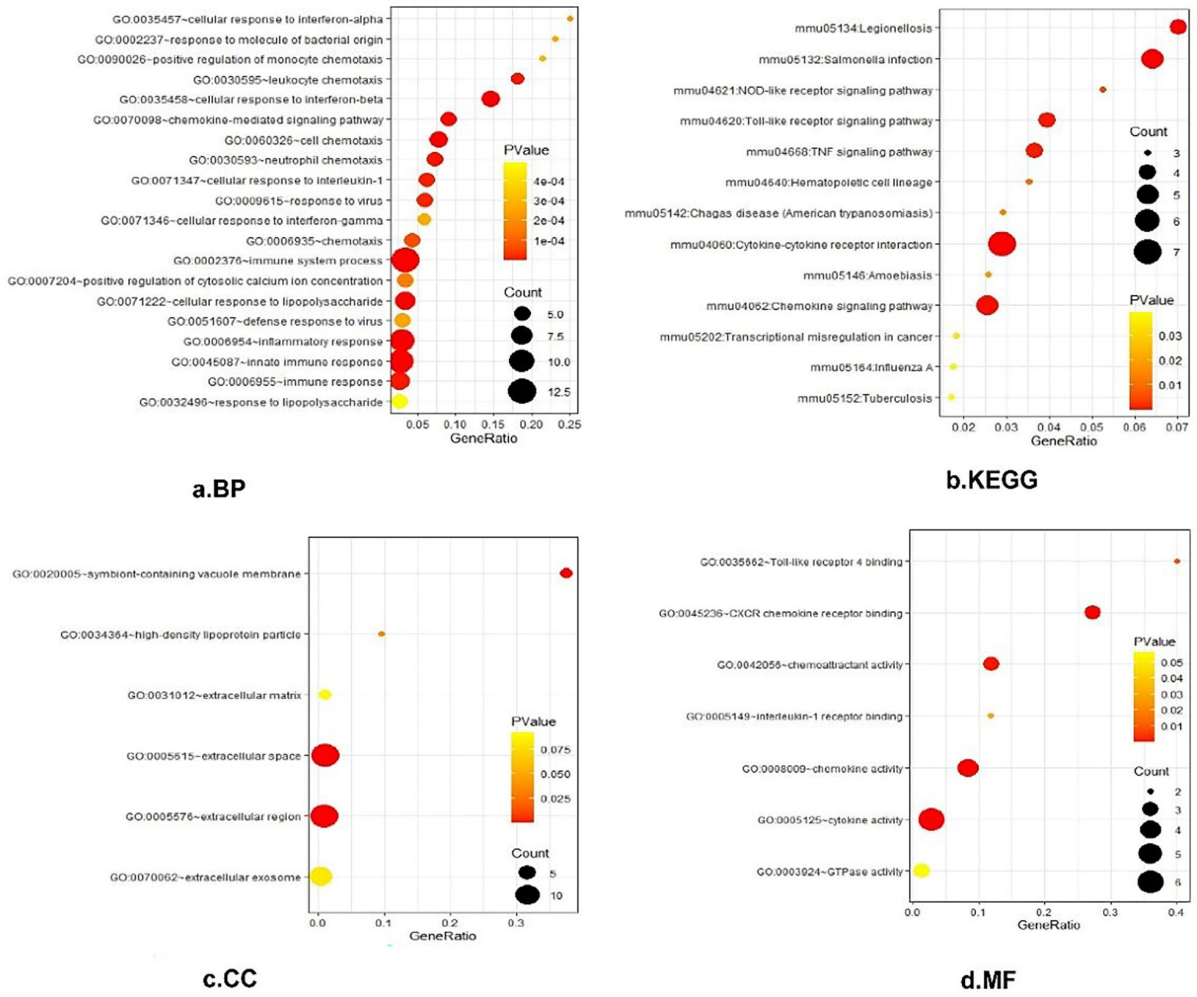
GO and KEGG enrichment analysis of DEGs. **a** Shows the results of biological process terms enriched by BP analysis. **b** Shows the enriched pathway by KEGG analysis. **c** Shows the results of biological process terms enriched by CC analysis. **d** Shows the results of biological process terms enriched by MF analysis. The coloured dots represent the *P*-value for that term, with red representing greater significance. The size of the dots represents the number of involved genes. The rich factor represents the proportion of enriched genes for each term

All 39 DEGs were further analyzed by DAVID software. The results of gene ontology analysis showed that (1) For biological process (BP), DEGs were particularly enriched in regulation of immune system process, innate immune response, inflammatory response, cellular response to interferon-beta and so on. (2) For cell component (CC), DEGs were mainly enriched in the extracellular space, the extracellular region, the symbiont-containing vacuole membrane, and the high-density lipoprotein particle. (3) For molecular function (MF), DEGs were enriched in the response to the cytokine activity, the chemokine activity, the CXCR chemokine receptor binding, the chemoattractant activity, the Toll-like receptor 4 binding and the interleukin-1 receptor binding (Fig. 2a, c, d). The analysis results of KEGG appeared that DEGs were enriched in multiple pathways (Fig. 2b), including cytokine–cytokine receptor interaction, Salmonella infection, Legionellosis, Chemokine signalling pathway, Toll-like receptor signalling pathway and so on (*P* < 0.05).

### Protein–protein interaction network (PPI) and modular analysis

**Fig. 3:**
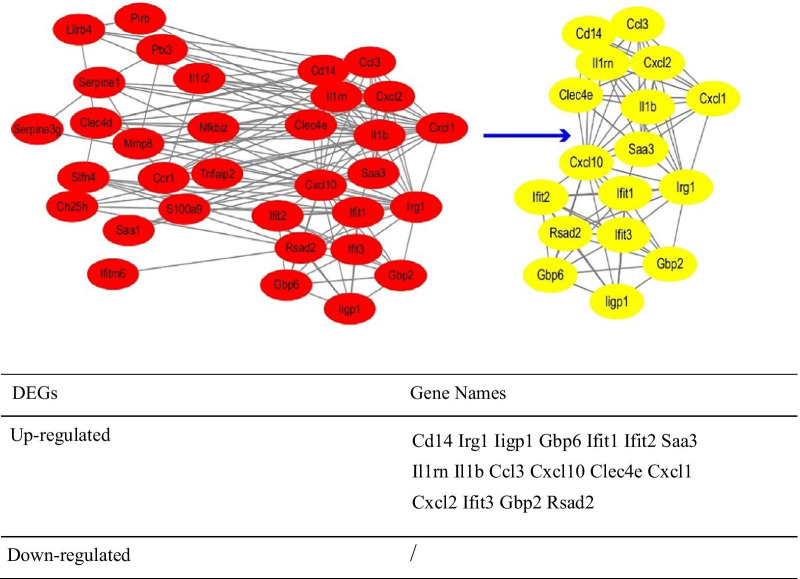
Common DEGs PPI network constructed by STRING online database and Module analysis. There was a total of 33 DEGs in the DEGs PPI network complex. The nodes meant proteins; the edges meant the interaction of proteins; blue circles meant down-regulated DEGs and red circles meant up-regulated DEGs. Module analysis via Cytoscape software (degree cutoff = 2, node score cutoff = 0.2, k-core = 2, and max.)

**Fig. 4:**
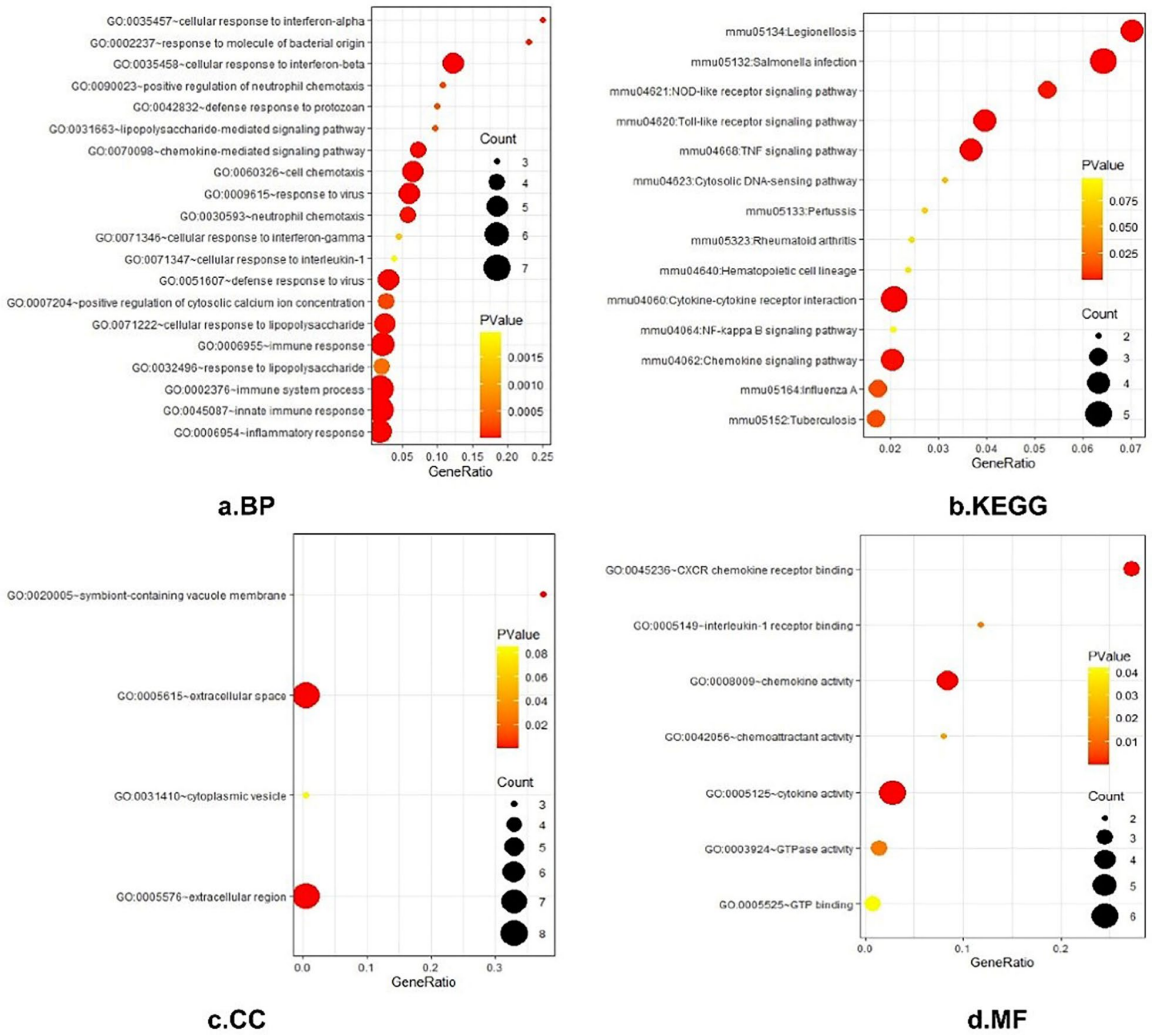
GO and KEGG enrichment re-analysis of DEGs. **a** Shows the results of biological process terms enriched by BP analysis. **b** Shows the enriched pathway by KEGG analysis. **c** Shows the results of biological process terms enriched by CC analysis. **d** Shows the results of biological process terms enriched by MF analysis. The coloured dots represent the *P*-value for that term, with red representing greater significance. The size of the dots represents the number of involved genes. The rich factor represents the proportion of enriched genes for each term

A total of 33 DEGs were imported into the DEGs PPI network complex which included 17 nodes and 77 edges, including 72 up-regulated and 0 down-regulated genes (Fig. 3). There were 16 genes excluded from the EDGs PPI network. Then Cytotype MCODE was applied for further analysis. It reviewed that 17 hub genes, including Cd14, Irg1, Iigp1, Gbp6, Ifit1, Ifit2, Saa3, Il1rn, Il1b, Ccl3, Cxcl10, Clec4e, Cxcl1, Cxcl2, Ifit3, Gbp2 and Rsad2, all of which were identified from the 33 nodes. Based on the PPI network analysis, GO term and KEGG pathway enrichment analysis was performed again. The result from GO enrichment analysis showed that hub genes were mostly enriched in the biological process (BP), including cellular response to interferon-beta and so on, in cell components (CC), including symbiont-containing vacuole membrane, extracellular space an extracellular region, and also enriched in the molecular function (MF), including cytokine activity and so on. KEGG pathway was mainly enriched in Salmonella infection signalling pathway and so on (Fig. 4). According to the biological process analysis, GBP6, GBP2, IFIT1, IFIT3 and IIGP1 were related to the cellular response to interferon-beta.

### The basic expression of hub genes in the lung and human other organs

**Fig. 5:**
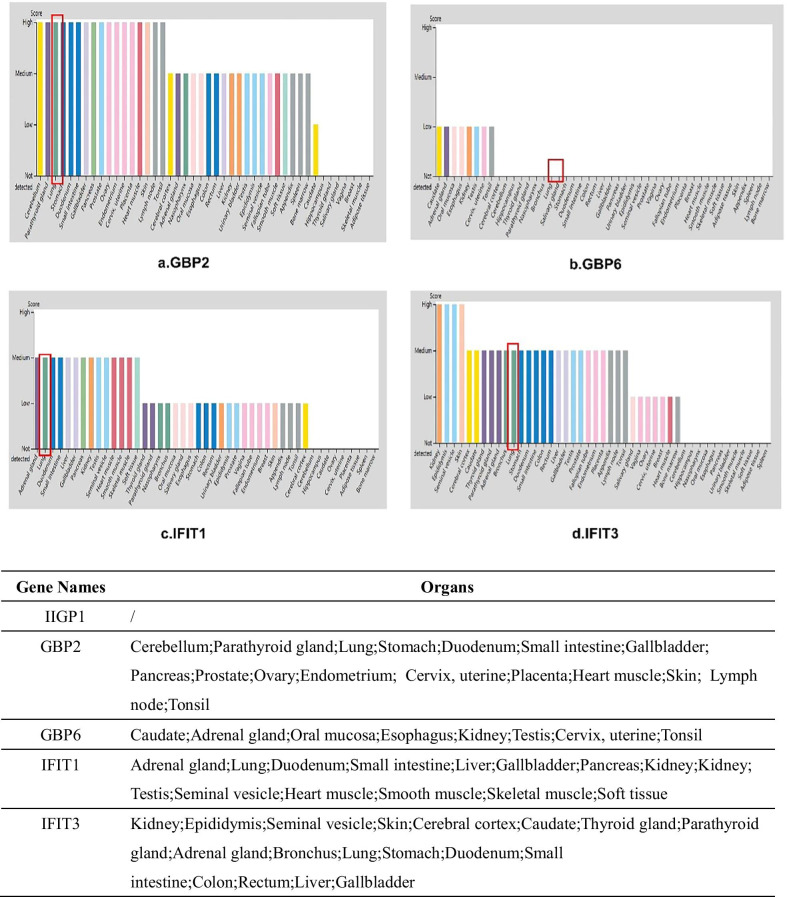
Basic expression of hub genes in the lung and other human organs via The Human Protein Atlas database. Protein expression level in different human organs, especially in the lung was evaluated with genotype-tissue expression (GTEx) project dataset from The Human Protein Atlas database

The Human Protein Atlas database was used to evaluate the expression level of cor- genes, including GBP6, GBP2, IFIT1, IFIT3 and IIGP1 in varied human organs. IIGP1 was mus musculus specific and not expressed in human. From Fig. 5a–d, GBP6, GBP2, IFIT1 and IFIT3 were expressed in multiple human organs with different expression levels in different tissues. But GBP6 were not detected in the human pulmonary tissue. It suggested that GBP2, IFIT1 and IFIT3 might be potential targets for ARDS diagnosis and treatment (Fig. 5).

### Transcription factor analysis of hub genes

**Fig. 6:**
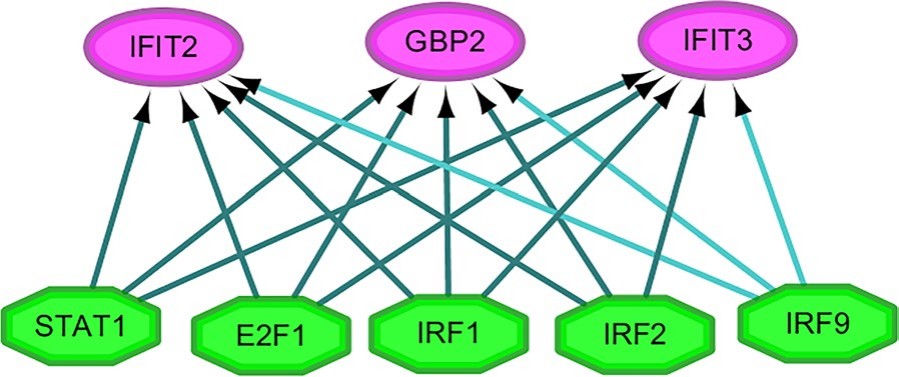
Common TFs among GBP2, IFIT2 and IFIT3 were screened by the iRegulon plugin of Cytoscape software. STAT1, E2F1, IRF1, IRF2, or IRF9 could modulate the three genes at the same time

The common transcription factor analysis of the 3 hub genes was conducted using iRegulon, a Cytoscape plugin, and a normalized enrichment shub (NES) > 12 was considered to be significant. The transcriptional regulation network of these hub genes was shown in Fig. 6. The transcription factors with NES > 12 were STAT1 (NES = 24.252), E2F1 (NES = 21.465), IRF2 (NES = 19.614), IRF1 (NES = 12.027) and IRF9 (NES = 12.007).

## Discussion

In the present study, a total of 39 genes related to ARDS were identified. GBP2, IFIT1 and IFIT3 were identified as common hub genes. They were mainly involved in Salmonella infection, cytokine–cytokine receptor interaction, TNF signalling pathway, Toll-like receptor signalling pathway and so on and were confirmed expressed in varied organs, including the lung tissue. STAT1, E2F1, IRF1, IRF2, and IRF9 were identified as the main TFs and were predicted to regulate these hub genes in ARDS.

IFIT1 and IFIT3 belong to the interferon-induced protein with the tetratricopeptide repeats (IFIT) protein family. They are involved in regulating immune responses and restrict viral infections through a variety of mechanisms, including the restriction of viral RNA translation [27]. Recent studies showed that IFIT3 could modulate IFIT1 RNA Binding specificity and protein stability [28, 29]. Xu et al. reported that IFIT3 transcription was dependent on NF-κB activation [30], while NF-κB played a vital role in ARDS [31, 32]. Exome-wide analysis showed that IFIT3 mutation was associated with COPD and airflow limitation [33]. All of them suggested that IFIT1 and IFIT3 mutation might be involved in the occurrence of ARDS and supported our hypothesis.

According to our findings, STAT1, E2F1, IRF1, IRF2, and IRF9 were screened as TFs according to iRegulon, which is the plugin of Cytoscape. STAT1 is a member of the STAT family of 7 cytoplasmic proteins. It had essential effects on innate immunity via defending the host from different infections [34]. Sevoflurane could reduce LPS-induced ARDS via modulating STAT1 [35]. In hepatocellular carcinoma, IFIT3 could bind signal transducer and activator of transcription 1 (STAT1) and STAT2 to enhance STAT1–STAT2 heterodimerization and nuclear translocation upon IFN-α treatment, thus promoting IFN-α effector signalling [36]. It suggested that the interaction between STAT1 and IFIT3 might play a significant role in ARDS progression. IRF1, IRF2, and IRF9 belong to the interferon regulatory factor (IRF) family. IRF-1 deficiency played a key role in the classical ROS-dependent release of NETs, which might serve as a novel target in ARDS [37]. In the recent COVID-19 studies, NETs contributed to COVID-19 related ARDS [38, 39] by contributing to excessive thrombosis. It suggested that IRF-1 might play a role in COVID-19 related ARDS. Wang et al. reported that LncRNA XIST could aggravate LPS-induced ARDS in mice by upregulating IRF2 [40]. The above reports were entirely consistent with our findings.

Despite our findings supported by some studies, we did not conduct further animal experiments and clinical data analysis to verify it. It gave us a hint for further study direction. Next, we will implement some animal experiments to develop more sensitive biomarkers and drugs, followed by some related clinical trials.

In summary, our bioinformatics analysis study identified three DEGs (GBP2, IFIT1 and IFIT3) in ARDS pulmonary tissues according to two different microarray datasets (GSE2411 and GSE130936). Results suggested that these three genes could be targets for the study of ARDS, and might be regulated by TFs, STAT1, E2F1, IRF1, IRF2, or IRF9. Anyway, these predictions would be verified by a series of experiments in the future. These studies have opened up new research directions for the diagnosis and treatment of ARDS.

## Conclusions

In this review, we used a bioinformatics approach to help explore genes, pathways and related TFs that might be involved in the occurrence of ARDS. We speculated GBP2, IFIT1 and IFIT3 might play important roles. The findings might provide novel insights into the development of promising targets for the diagnosis and treatment of ARDS in the future.

## Supplementary Information

**Additional file 1. Supplementary Fig. 1**. DEGs gene ontology and KEGG pathway analysis by Enrichr software.

## Data Availability

The datasets generated and analysed during the current study are available in the GEO datasets repository, (https://www.ncbi.nlm.nih.gov/geo/). The datasets generated and analysed during the current study are available in the STRING repository, (https://string-db.org/).
