# Supplementary material for: Identification of significant alteration genes, pathways and TFs induced by LPS in ARDS via bioinformatical analysis
Source: BMC Infect Dis. 2021 Aug 21;21:852. doi: 10.1186/s12879-021-06578-7 (PMC8379573; doi:10.1186/s12879-021-06578-7)
Supplement: Supplementary file 1 — Additional file 1. Supplementary Fig. 1. DEGs gene ontology and KEGG pathway analysis by Enrichr software. [file 12879_2021_6578_MOESM1_ESM.docx]

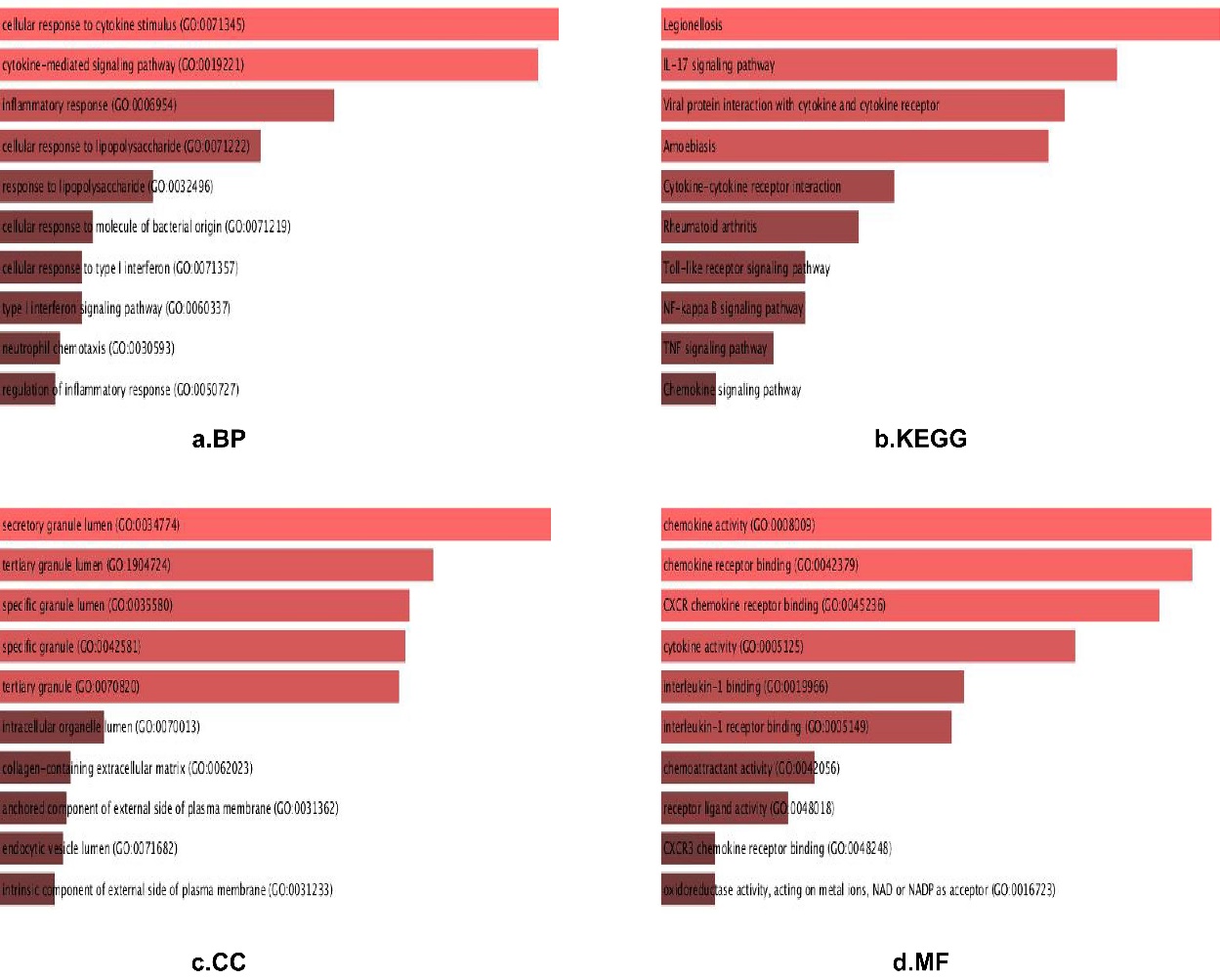
***Supplementary Fig.1:* DEGs gene ontology and KEGG pathway analysis by Enrichr software**

(a) Shows the results of biological process terms enriched by BP analysis. (b) Shows the enriched pathway by KEGG analysis. (c) Shows the results of biological process terms enriched by CC analysis. (d) Shows the results of biological process terms enriched by MF analysis. The coloured bands represent the *P*-value for that term, with red representing greater significance. The length of bands represents the number of enriched genes.

All 39 DEGs were further analyzed by Enrichr software (<https://maayanlab.cloud/Enrichr/>). The results of gene ontology analysis showed that a) For biological process (BP), DEGs were particularly enriched in regulation of cellular response to cytokine stimulus, cytokine-mediated signalling pathway, inflammatory response, cellular response to lipopolysaccharide and so on. c) For cell component (CC), DEGs were mainly enriched in secretory granule lumen, tertiary granule lumen and so on. d) For molecular function (MF), DEGs were enriched in the chemokine activity, chemokine receptor binding, CXCR chemokine receptor binding, cytokine activity and so on (FigureS a, c, d). b)The analysis results of KEGG appeared that DEGs were enriched in multiple pathways (FigureS b), including Cytokine-cytokine receptor interaction, IL-17 signalling pathway, Viral protein interaction with cytokine and cytokine receptor and so on (*p*<0.05).
